# Downregulation of organic cation transporters OCT1 (*SLC22A1*) and OCT3 (*SLC22A3*) in human hepatocellular carcinoma and their prognostic significance

**DOI:** 10.1186/1471-2407-12-109

**Published:** 2012-03-22

**Authors:** Michael Heise, Anja Lautem, Johanna Knapstein, Jörn M Schattenberg, Maria Hoppe-Lotichius, Daniel Foltys, Nina Weiler, Anca Zimmermann, Arno Schad, Dirk Gründemann, Gerd Otto, Peter R Galle, Marcus Schuchmann, Tim Zimmermann

**Affiliations:** 1 1st Department of Internal Medicine, Johannes Gutenberg University Mainz, Germany; 2 Department of Hepatobiliary and Transplantation Surgery, Johannes Gutenberg University Mainz, Germany; 3 Department of Pharmacology, University of Cologne, Germany; 4 Institute of Pathology, Johannes Gutenberg University Mainz, Germany

**Keywords:** OCT1, OCT3, SLC22A1, SLC22A3, Hepatocellular carcinoma, HCC

## Abstract

**Background:**

Organic cation transporters (OCT) are responsible for the uptake and intracellular inactivation of a broad spectrum of endogenous substrates and detoxification of xenobiotics and chemotherapeutics. The transporters became pharmaceutically interesting, because OCTs are determinants of the cytotoxicity of platin derivates and the transport activity has been shown to correlate with the sensitivity of tumors towards tyrosine kinase inhibitors. No data exist about the relevance of OCTs in hepatocellular carcinoma (HCC).

**Methods:**

OCT1 (*SLC22A1*) and OCT3 (*SLC22A3*) mRNA expression was measured in primary human HCC and corresponding non neoplastic tumor surrounding tissue (TST) by real time PCR (n = 53). Protein expression was determined by western blot analysis and immunofluorescence. Data were correlated with the clinicopathological parameters of HCCs.

**Results:**

Real time PCR showed a downregulation of *SLC22A1 *and *SLC22A3 *in HCC compared to TST (p ≤ 0.001). A low *SLC22A1 *expression was associated with a worse patient survival (p < 0.05). Downregulation was significantly associated with advanced HCC stages, indicated by a higher number of T3 tumors (p = 0.025) with a larger tumor diameter (p = 0.035), a worse differentiation (p = 0.001) and higher AFP-levels (p = 0.019). In accordance, *SLC22A1 *was less frequently downregulated in tumors with lower stages who underwent transarterial chemoembolization (p < 0.001) and liver transplantation (p = 0.001). Tumors with a low *SLC22A1 *expression (< median) showed a higher *SLC22A3 *expression compared to HCC with high *SLC22A1 *expression (p < 0.001). However, there was no significant difference in tumor characteristics according to the level of the *SLC22A3 *expression.

In the western blot analysis we found a different protein expression pattern in tumor samples with a more diffuse staining in the immunofluorescence suggesting that especially OCT1 is not functional in advanced HCC.

**Conclusion:**

The downregulation of OCT1 is associated with tumor progression and a worse patient survival.

## Background

Hepatocellular carcinoma (HCC) is one of the most common human malignancies worldwide with growing numbers of patients frequently leading to death [1]. In the majority of cases tumors grow multifocal and show a high rate of recurrence with a poor prognosis [2]. In general, HCCs do not respond to classical chemotherapeutics. Treatment options, especially with regard to a systemic therapy, are very limited. Therefore, new markers and therapeutic usable targets are urgently needed.

Transporters contribute to the survival of a single cell in a higher organism and mediate the interaction between cells and their environment. Moreover, they play an important role in the cellular uptake of anticancer drugs and the development of multidrug resistance.

The three organic cation transporters OCT 1 (*SLC22A1*), OCT2 (*SLC22A2*) and OCT3 (synonym: extraneuronal monoamine transporter--EMT) (*SLC22A3*) belong to the amphiphilic solute facilitator (ASF) family of integral transmembrane proteins and are involved in many metabolic processes and detoxification.

The role of OCTs in metabolism is the uptake, intracellular inactivation and biliary or urinary excretion of a broad spectrum of endogenous (e.g. catecholamines) and exogenous substrates (e.g. metformine, betablockers, etc.) as well as anticancer drugs (e.g. platin derivatives) [3-11].

Therefore, OCTs became pharmaceutically interesting: the OCT1 activity was reported to correlate with the sensitivity of tyrosine kinase inhibitors (TKI), e.g. imatinib, in patients with chronic myeloid leukemia (CML) [12-14]. Furthermore, OCTs are determinants of the cytotoxicity of platin derivates, which is relevant for the responsiveness towards platin-containing chemotherapies [11].

Since important metabolic pathways are secured, the OCTs have overlapping substrate specificities and tissue expression patterns. In human OCT1 is mainly expressed in the liver, OCT 2 in the kidney, whereas OCT3 is widely distributed in many tissues.

Interestingly, OCT1 was reported to be not relevantly expressed in liver cancer cell lines [15]. Therefore, the question arose whether OCT1 and OCT3, which are physiologically expressed in the liver, are present in primary HCC.

The aim of our study was to elucidate the impact of OCT expression on HCC and patient survival. Expression levels were measured in HCC and corresponding non neoplastic tumor surrounding tissue (TST) and correlated with clinicopathological parameters and outcomes.

## Methods

### Patient tissue samples

Normal liver tissue was obtained from 10 patients who underwent liver biopsy for elevated liver enzymes but showed completely normal histology. HCC tumor samples and corresponding TST were obtained from 53 patients undergoing liver resection or liver transplantation (LT) between 2006 and 2011 at the Department of Hepatobiliary and Transplantation Surgery of the Johannes Gutenberg University Mainz, Germany. Informed consent was given by each patient. The study followed the ethical guidelines of the Declaration of Helsinki and was approved by the local ethics committee. Liver tissues were immediately shock frozen after resection and underwent liquid nitrogen storage prior to analysis. All HCC were histological confirmed.

### Culture of tumor cell lines

Hepatic tumor cell lines (HepG2, Hep3b, Huh6 and Huh7) were cultured in DMEM/GlutaMax-1 (InVitrogen, Carlsbad, CA, USA) supplemented with 10% FCS (PAA, Pasching, Austria) and 1% penicillin/streptomycin (Cambrex, East Rutherford, NJ, USA). Cells were grown at 37°C in 5% CO_2 _atmosphere in cell culture flasks and medium was replaced every 2 days. Prior to mRNA isolation, cells were removed from the flasks by 0.05% trypsin/EDTA (PAA, Pasching, Austria) treatment. HEK 293 cells stably transfected with pcDNA3 OCT1 and pcDNA3 OCT3 constructs were kindly provided by Dirk Gründemann and served as positive control.

### RNA isolation, RT-PCR and real-time RT-PCR analysis

RNA isolation of tumor cell lines was done using the RNeasy Mini Kit (Qiagen, Hilden, Germany) according to the manufacturer's recommendations. RNA from tissue samples was extracted by the High Pure RNA Tissue Kit (Roche, Mannheim, Germany). cDNA preparation from total RNA was performed with 500 ng RNA (20 μl total volume) using the iScript cDNA Synthesis kit (Biorad, München, Germany). All kits mentioned were used according to the manufacturer's recommendations. For semiquantitative RT-PCR analysis 1 μl (200 ng) of the cDNA was used as template for the specific PCRs using Red Taq Ready Mix PCR Reaction mix (Sigma, Munich, Germany). Primers applied were GAPDH: forward 5'-AAT CCC ATC ACC ATC TTC CA-3', reverse 5'-TGT GGT CAT GAG TCC TTC CA-3' (318 bp fragment); OCT1 (SLC22A1): forward 5'-GTG TGT AGA CCC CCT GGC TA-3', reverse 5'-GTG TAG CCA GCC ATC CAG TT-3' (363 bp fragment); OCT3 (SLC22A3): forward 5'-ATC GTC AGC GAG TTT GAC CT-3', reverse 5'-TTG AAT CAC GAT TCC CAC AA-3' (324 bp fragment). Cycling conditions were as follows: initial denaturation (3 min 95°C) followed by 36 cycles of denaturation (30s at 94°C), annealing (1 min at 60°C), and elongation (1 min at 72°C). After the last cycle, a final extension (10 min at 72°C) was added, and thereafter, the samples were kept at 4°C. 20 μl of the products were run on a 1.5% agarose gel stained by ethidium bromide, and analyzed under UV light.

Quantitative analysis of OCT1 (*SLC22A1*) and OCT3 (*SLC22A3*) transcripts was performed by real-time RT-PCR. The Quantitect SYBR Green PCR Kit (Qiagen, Hilden, Germany) and validated primers of a Quantitect Primer Assay with the primer sets HS_SLC22A1_1_SC (OCT1; 120 bp fragment), HS_SLC220A3_1_SC (OCT3; 115 bp fragment) and HS_GAPDH_2_SG (GAPDH; 119 bp fragment) (Qiagen, Hilden, Germany) were used according to the manufacturers instructions. For the amplification, an initial denaturation at 95°C for 15 min, followed by 15 s at 94°C, 30s at 55°C and 30s at 72°C for 40 cycles was used. Samples were run on a LightCycler^® ^480 real-time PCR system (Roche, Mannheim, Germany). The relative expression levels of OCT1 (*SLC22A1*) and OCT3 (*SLC22A3*) mRNA in HCC and TST were calculated by normalization to GAPDH gene expression using the LightCycler^® ^480 software Release 1.5.0. For examination of OCT1 (*SLC22A1*) and OCT3 (*SLC22A3*) mRNA regulation, the relative mRNA expression of tumor tissue (HCC) was related to the relative mRNA expression of corresponding TST. The median expression of this ratio (SLC22A1: 0.112; SLC22A3: 0.658) was used to define a cut-off value to subdivide tumor tissues into high or low expressing HCCs.

### Western blot analysis

Total protein extracts were prepared in sample buffer pH 8.0 containing 20 mM Tris, 5 mM EDTA, 0.5% TritonX-100 and complete Mini, EDTA-free protease inhibitors (1:25; Roche Diagnostics, Germany). For Western blot analyses 60 μg of protein were loaded on a 12% SDS-PAGE gel. The gel was transferred onto a nitrocellulose transfer membrane (OPTITRAN BA-S85/Whatman) following separation. Mouse-anti-OCT1 monoclonal antibody (1:1000; Novus Biologicals, Littleton, CO, USA), rabbit-anti-OCT3 (1:2000; Lifespan Biosciences, Seattle, WA, USA) polyclonal antiserum or goat-anti-actin (1:10000; Santa Cruz Biotechnology, Santa Cruz, CA, USA) polyclonal antiserum were used as primary antibody. Horseradish peroxidase (HRP)-conjugated anti-mouse, anti-rabbit or anti-goat IgG (DAKO Cytomation, Hamburg, Germany) were used as secondary antibody at 1:10000 dilution. Protein bands were visualized using Western Lightning^® ^Plus-ECL, enhanced chemiluminescent substrate (Perkin Elmer, Waltham, MA, USA).

### Immunofluorescence

The localization of OCT1 in human HCC samples and corresponding TST was analyzed on frozen sections by immunofluorescence microscopy. Prior to staining sections were fixed for 20 min with 4% paraformaldehyde/PBS at room temperature in a wet chamber. After fixation, cells were washed with TBS (pH 7.6)/0.1% Tween. After preincubation with hydrogen peroxide for blocking of endogenous peroxidase, endogenous biotin was blocked with the Avidin-Biotin Blocking kit (Vector Laboratories, Burlingame, CA, USA) and contaminating proteins were inhibited by ROTI^®^-Immunoblock solution (ROTH, Karlsuhe, Germany). Then, samples were incubated with mouse-anti-human OCT1 (1:200; Novus Biologicals, Littleton, CO, USA) as primary antibody. After incubation with the secondary antibody (goat anti-rabbit IgG-Biotin, 1:1000; DAKO Cytomation, Hamburg, Germany), the TSA™ Cyanine system (Perkin Elmer, Waltham, MA, USA) was added. For negative control the primary antibody was omitted. The images were evaluated under a fluorescence microscope (Olympus BX51, Olympus U-RFL-T).

### Statistical analysis

Data management and all statistical analyses were performed with the SPSS program (IBM^® ^SPSS^® ^Version 19.0). For categorical variables, between-group differences were analyzed by the chi-square or Fisher's exact test. For quantitative variables data were expressed as median and range. If distribution was normal and sample sizes tested sufficient, equality of variances was analyzed with the Levene's test and samples were compared using the paired t-test. If variables were not normally distributed or sample size was too small, we applied the Wilcoxon rank sum nonparametric test. All tests were performed using a five percent level of significance (two-sided). Overall survival rates were calculated using the Kaplan-Meier method and compared using the log-rank test.

## Results

### Expression of OCT1 (*SLC22A1*) and OCT3 (*SLC22A3*) mRNA in liver cancer cell lines, normal liver and primary HCCs

**Figure 1:**
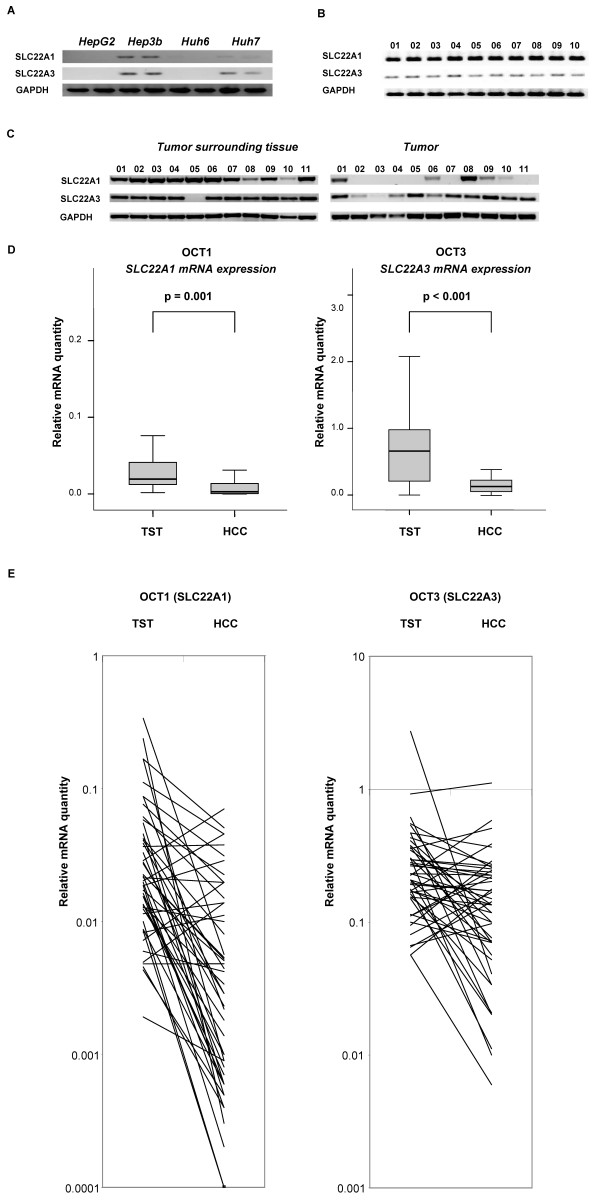
**(A) mRNA expression (RT-PCR) of OCT1 (*SLC22A1*) and OCT3 (*SLC22A3*) in hepatic tumor cell lines (HepG2, Hep3b, Huh6, Huh7) relative to glyceraldehyde-3-phosphate dehydrogenase (GAPDH)**. (**B**) mRNA expression (RT-PCR) of OCT1 (*SLC22A1*) and OCT3 (*SLC22A3*) in normal liver tissue of 10 patients relative to GAPDH. (**C**) Exemplarily mRNA expression (RT-PCR) in eleven patients (1-11) with HCC and the corresponding non neoplastic tumor surrounding tissue (TST). (**D**) Downregulation of OCT1 (*SLC22A1*) and OCT3 (*SLC22A3*) mRNA expression in HCC (n = 53) compared to TST. (**E**) Individual OCT1 (*SLC22A1*) and OCT3 (*SLC22A3*) mRNA expression pattern in each patient (HCC and according TST).

To analyse the role of OCT1 and OCT3 in malignant liver cells, we first studied the mRNA expression in four different human liver cancer cell lines (HepG2, Hep3b, Huh6, Huh7). RT-PCR using glyceraldehyde-3-phosphate dehydrogenase (GAPDH)-normalized cDNAs revealed that *SLC22A1 *and *SLC22A3 *transcripts were completely absent in HepG2 and Huh6 cell lines and only low expression levels could be detected in Hep3b and Huh7 cell lines (Figure 1A). *SLC22A1 *mRNA was almost absent in Huh7 cell lines. Therefore, these tumor cell lines seemed not to be suited for the investigation of *SLC22A1 *and *SLC22A3 *expression in vitro.

Then we investigated the mRNA expression of *SLC22A1 *and *SLC22A3 *in healthy normal human liver (n = 10), HCC (n = 53) and corresponding TST. Normal liver served as control for the expression of *SLC22A1 *and *SLC22A3*. Figure 1B representatively shows the RT-PCR results of 10 normal and healthy liver control samples. As expected, *SLC22A1 *mRNA was strongly expressed in human liver tissue, whereas *SLC22A3 *mRNA was expressed to a lower degree. TST exhibited comparable expression of *SLC22A1 *and *SLC22A3 *mRNA with normal liver tissue. Differences in the expression between TST and cancerous tissue of 11 patients are exemplarily shown in Figure 1C.

Real time data confirmed a strong downregulation of *SLC22A1 *mRNA expression in HCC compared to corresponding TST (p = 0.001; Figure 1D). *SLC22A3 *was also found to be downregulated in primary HCC compared to TST (p < 0.001). Individual expression patterns in HCC and the corresponding TST are demonstrated in Figure 1E. Details of clinical and pathological characteristics of the patients and tumors were summarized in Table 1 according to WHO specifications. Compared to TST *SLC22A1 *mRNA was in average 2,3-fold (± 1,4-fold) down-regulated in tumor tissue of most HCCs, whereas downregulation of *SLC22A3 *mRNA was lower (1,2-fold (± 1,2-fold)) (Figure 1C, D, E).

**Table 1:** Patients and tumor characteristics

Characteristics	
n	53

Median follow-up in days (range)	434 (22-1645)

Male/female (n)	46/7

Median age in years (range)	67 (35-93)

1-3 nodules/multiple nodules (specimen)	36/17

Tumor diameter < 3 cm/> 3 cm	19/34

Median tumor diameter in cm (range)	5.0 (0.1-30)

T classification: T1/T2/T3 (specimen)	18/20/15

Grading: G1/G2/G3/Gx	11/31/10/1

AFP > 100/< 100 *	11/40

Angioinvasion yes/no ^#^	11/38

Child A/B/C/no cirrhosis	19/4/7/23

Underlying disease (HCV/HBV/alcoholic/others/unknown)	10/9/ 4/15/15

Pretreatment with chemoembolization yes/no	20/33

Liver transplantation/resection	20/33

* 2 unknown ^# ^4 unknown

### Reduced survival in patients with intratumoral OCT1 (*SLC22A1*) downregulation

According to the real time PCR results, we divided the patients into two groups: a high expression group (expression level ≥ median) and a low expression group (expression level < median).

**Figure 2:**
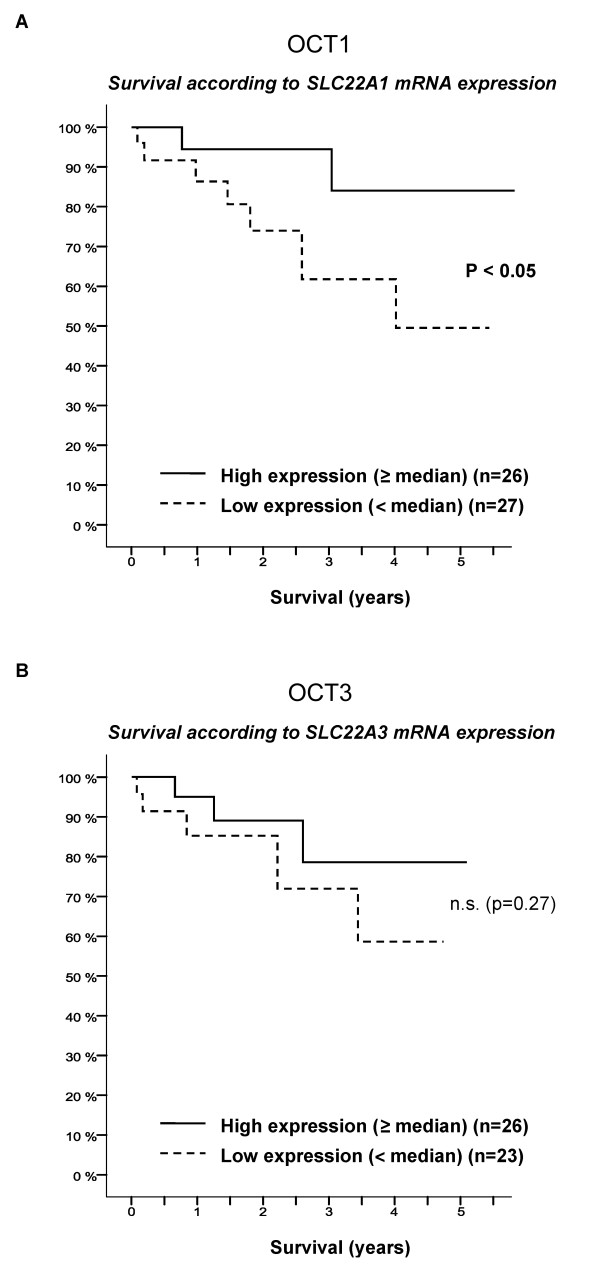
**(A) Survival of HCC patients according to the intratumoral OCT1 (*SLC22A1*) mRNA expression (real time data)**. Downregulation of OCT1 is associated with a significantly worse patient survival (p < 0.05). (**B**) Survival of HCC patients according to the intratumoral OCT3 (*SLC22A3*) mRNA expression (real time data).

Considering the *SLC22A1 *mRNA expression, there was a significantly reduced overall patient survival in patients with a low *SLC22A1 *mRNA expression in HCC (p < 0.05) (Figure 2A). Although OCT3 (*SLC22A3*) mRNA expression was also downregulated (Figure 1D), the overall survival rate of HCC patients with low expression of *SLC22A3 *mRNA was not significantly different from that of patients with high *SLC22A3 *mRNA expression (p = 0.27; Figure 2B).

### Patient and tumor characteristics according to the OCT1 (*SLC22A1*) mRNA expression

Low OCT1 (*SLC22A1*) mRNA expression levels were associated with advanced HCC stages as indicated by a higher frequency of T3 carcinomas (p = 0.025) with a larger tumor diameter (p = 0.035), a worse tissue differentiation as indicated by grading (p = 0.001) and higher AFP-levels (p = 0.019). Tumor characteristics according to the *SLC22A1 *mRNA expression are shown in Table 2. *SLC22A1 *was less frequently downregulated in tumors with lower stages who underwent transarterial chemoembolization (p < 0.001) and liver transplantation (p = 0.001). Tumors with a low *SLC22A1 *expression (< median) showed a higher *SLC22A3 *expression compared to HCC with high *SLC22A1 *expression (p < 0.001). However, there was no significance in tumor characteristics for the *SLC22A3 *expression (Table 3).

**Table 2:** Patients and tumor characteristics in dependence of the intratumoral OCT1 mRNA expression

Characteristics	OCT1 (*SLC22A1*) Low expression (< Median)	OCT1 (*SLC22A1*) High expression (≥ Median)	P-value
N	27	26	

Median follow-up in days (range)	434 (22-1538)	425 (109-1645)	n.s. (0.20)

Male/female (n)	23/4	23/3	n.s. (1.00)

Median age in years (range)	69 (35-86)	67 (47-93)	n.s. (0.35)

1-3 nodules/multiple nodules (specimen)	18/9	18/8	n.s. (1.00)

Tumor diameter < 3 cm/> 3 cm	5/22	14/12	**0.010**

Median tumor diameter in cm (range)	6.0 (1.5-30)	2.8 (0.1-20)	**0.035**

T classification: T1/T2/T3 (specimen)	8/7/12	10/13/3	**0.025**

Grading: G1/G2/G3/Gx	1/17/9/0	10/14/1/1	**0.001**

AFP > 100/< 100 *	9/16	2/24	**0.019**

Angioinvasion yes/no ^#^	7/16	4/22	n.s. (0.31)

Child A/B/C/no cirrhosis	10/1/1/15	9/3/6/8	n.s. (0.08)

Underlying disease (HCV/HBV/alcoholic/others/unknown)	4/2/1/10/10	6/7/3/5/5	n.s. (0.11)

*SLC22A3 *mRNA expression ratio HCC/TST (range)	0.21 (0.001-3.07)	0.86 (0.33-3.12)	**0.008**

Pretreatment with chemoembolization (yes/no)	2/25	18/8	**< 0.001**

Liver transplantation/resection	4/23	16/10	**0.001**

Median: 0.112 * 2 unknown ^# ^4 unknown

**Table 3:** Patients and tumor characteristics in dependence of the intratumoral OCT3 mRNA expression

Characteristics	OCT3 (*SLC22A3*) Low expression (< Median)	OCT3 (*SLC22A3*) High expression (≥ Median)	P-value
N	23	26	

Median follow-up in days (range)	348 (22-1531)	423 (29-1645)	n.s. (0.36)

Male/female (n)	21/2	22/4	n.s. (0.67)

Median age in years (range)	71 (35-84)	64 (47-93)	n.s. (0.13)

1-3 nodules/multiple nodules (specimen)	16/7	18/8	n.s. (0.98)

Tumor diameter < 3 cm/> 3 cm	7/16	11/15	n.s. (0.55)

Median tumor diameter in cm (range)	5.0 (1.5-30)	4.4 (0.1-20)	n.s. (0.98)

T classification: T1/T2/T3 (specimen)	6/9/8	10/11/5	n.s. (0.42)

Grading: G1/G2/G3/Gx	3/15/5/0	6/15/4/1	n.s. (0.60)

AFP > 100/< 100 *	4/17	7/19	n.s. (0.73)

Angioinvasion yes/no ^#^	3/18	7/17	n.s. (0.30)

Child A/B/C/no cirrhosis	11/0/1/11	7/4/5/10	n.s. (0.06)

Underlying disease (HCV/HBV/alcoholic/others/unknown)	5/2/2/6/8	4/6/2/8/6	n.s. (0.64)

*SLC22A1 *mRNA expression ratio HCC/TST (range)	0.08 (0.001-1.36)	0.4 (0.003-2.78)	**0.008**

Pretreatment with chemoembolization (yes/no)	6/17	14/12	n.s. (0.08)

Liver transplantation/resection	6/17	13/13	n.s. (0.14)

Median: 0.658 * 2 unknown ^# ^4 unknown

### Protein expression of OCT1 in human HCCs

**Figure 3:**
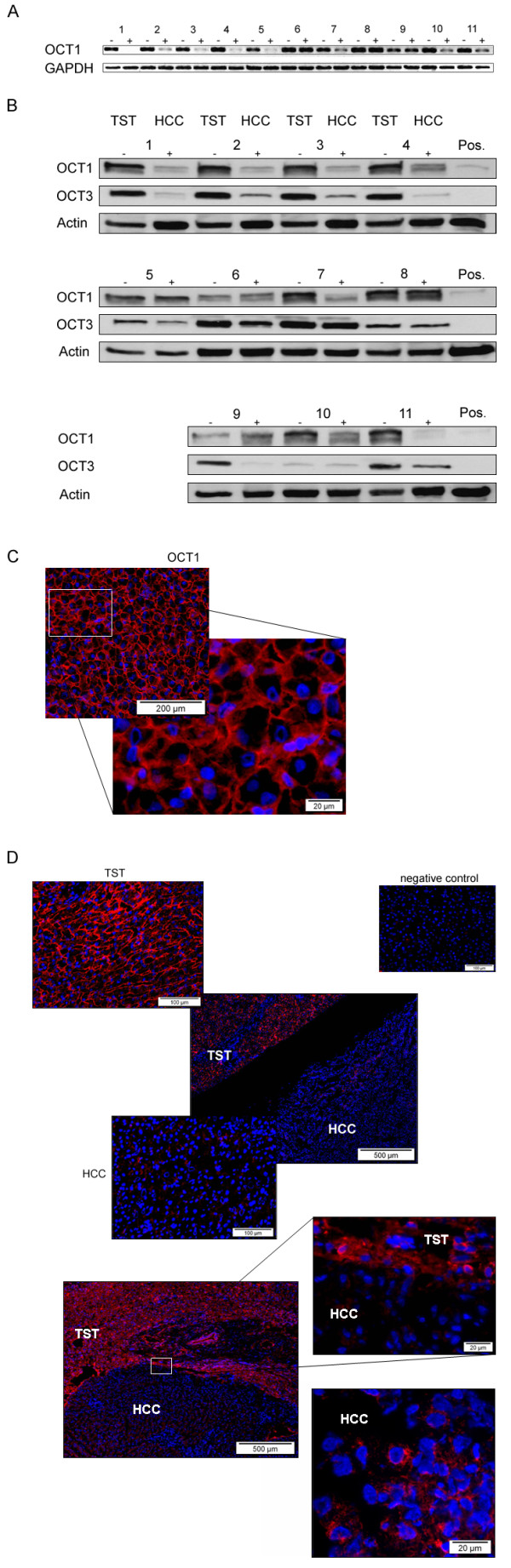
**(A) RT-PCR analysis (- TST; + HCC) and Western blot analysis (B) of eleven patients with HCC and the corresponding TST**. Protein expression and downregulation of the transporters, especially OCT1, correlate well compared with the RT-PCR data. Stably transfected cancer cell lines (pcDNA3_OCT1h) served as positive control for OCT1 (Pos.); (OCT1: 61kD; OCT3: 60kD). **(C) Analysis of OCT1 protein expression by immunofluorescence**. OCT1 shows a membranous staining in normal liver tissue, whereas the protein is downregulated in HCC **(D)**. Tumor borders of two cases with downregulation of OCT1 in HCC (upper panel: case 2--Figure 3A/3B; lower panel: case 3--Figure 3A/3B). There is a diffuse staining near the tumor border as well as in the tumor tissue (lower panel) suggesting that the transporter function is affected in HCC.

To examine the protein expression of the transporters in HCC we performed western blot analysis. Figure 3 shows that the downregulation of the transporters, especially OCT1, as measured in the RT-PCR (Figure 3A) correlates well with the corresponding protein levels (Figure 3B). Because of the impact of OCT1 on tumor characteristics and patient survival we focused on this transporter. To identify the localization of OCT1 in tumor tissue, we subsequently evaluated protein expression by fluorescence microscopy (Figure 3C, 3D). In normal liver we detected a membranous staining (Figure 3C). In contrast, in HCC OCT1 protein was not predominantly localized on the cell membrane of cancerous hepatocytes, but was also present in the cytoplasm (Figure 3D), suggesting the presence of non-functional OCT1 protein with abrogated transport function in tumor tissue.

## Discussion

OCTs have been extensively studied in many tissues and tumor cell lines, but only few data exist about the presence and their role in human malignancies. In colorectal cancer, Zhang et al. found a mRNA expression of OCT2 (*SLC22A2*) in tumor samples, whereas this transporter was not expressed in normal colon tissue [11]. SNPs in OCT3 (*SLC22A3*) were reported to contribute to the risk of distal colon cancer in an Asian population [16].

To our knowledge, this is the first study analyzing the expression profiles of the OCTs in a larger series of human hepatocellular carcinomas with direct correlation to clinical and tumor-specific data.

The human HCC samples analyzed, revealed different intensities of OCT1 (*SLC22A1*) and OCT3 (*SLC22A3*) expression ranging from absent to strong. We did not examine OCT2 in light of the absent to very rare and low expression in the human liver. Expression rates in RT-PCR were too low to establish meaningful statistical results.

We showed a significant downregulation for both transporters in HCC compared to the corresponding TST (p ≤ 0.001). Downregulation of *SLC22A1 *mRNA expression in primary human HCC is in line with previously published microarray findings by Park et al., where *SLC22A1 *showed a significantly reduced expression in HCC [17].

Our results provide first evidence that the downregulation of *SLC22A1 *mRNA expression is associated with advanced tumor stages and worse patient survival (Table 2 Figure 2A). Furthermore, these tumors had significantly higher T values (p = 0.025), G values (p = 0.001) and median tumor diameters (p = 0.035, Table 2). In tumors with lower stages, who underwent transarterial chemoembolization and liver transplantation, *SLC22A1 *was less frequently downregulated (p < 0.001). In our center transarterial chemoembolization is performed with lipiodol (20 mL) and mitomycin c (10 mg in 20 mL water) every six weeks until transplantation. Both substances are not known to be substrates for OCTs. The response to transarterial chemoembolization is discussed to represent a biological selection criterion for patients with HCC during the waiting time for liver transplantation [18]. Whether OCT1 might be a molecular marker involved in mechanisms influencing the response to transarterial chemoembolization or a marker to predict the tumor biology and the risk of recurrence after liver transplantation remains to be determined.

Interestingly, tumors with a low *SLC22A1 *expression showed a higher *SLC22A3 *expression compared to HCC with high *SLC22A1 *expression (p < 0.001), which is in contrast to the physiological state with high *SLC22A1 *and low *SLC22A3 *expression. The importance of these findings, especially with regard to tumor therapy needs to be defined in future.

Although OCTs are functionally influenced by many endogenous and exogenous substances, to date there are no data about pathways and mechanisms of the regulation of OCT expression in cancer. The transporters are located on the chromosomal locus 6q26-27 in a conserved gene cluster and influenced by genomic imprinting mechanisms involving the well known IGF2R tumor suppressor gene [19,20]. This may contribute to complex genetic and epigenetic regulations in the context of human malignancy.

Interestingly, the OCTs could also be directly associated with mechanisms of tumor development by influence of environmental factors and carcinogens. In HepG2 cells expression of *SLC22A3 *correlates with the carcinogenic potency after treatment with polycyclic aromatic hydrocarbons (PAH), which are known to be potential carcinogens [21].

Furthermore, OCTs may play an important role in the therapy of malignant tumors. They are held responsible for the cytotoxicity of platin derivatives and are predictors of responses to small molecules [11,22-24]. Downregulation in HCC could be responsible for a worse or lacking response towards platin treatment. In colorectal cancer OCTs are determinants of oxaliplatin cytotoxicity [11,25-27]. Moreover, *SLC22A3 *expression in renal cell carcinoma cell lines enhances the sensitivity towards chemotherapeutics as melphalan, irinotecan and vincristin [28].

In patients with chronic myeloid leukaemia *SLC22A1 *expression is associated with treatment response to the tyrosine kinase inhibitor (TKI) imatinib [13,22,29]. Another multi-tyrosine kinase inhibitor sorafenib, which is used as systemic therapy in HCC, reduces the cellular uptake of platin and the platin-induced cytotoxicity in human colorectal cancer cell lines [30]. Recently, it has been shown, that oxaliplatin uptake in CRC is attenuated by the TKI saracatinib via inhibition of OCT2 [31]. These findings elucidate the crucial role of OCTs in different treatment protocols affecting classical chemotherapeutics as platin derivatives as well as small molecules like TKI. It is still unknown whether the presence of OCTs in tumors is beneficial with regard to therapy or deleterious by supporting tumor growth. Our data suggest, that a downregulation of OCT1 in the liver with possibly lacking function is associated with a worse outcome regardless of TKI treatment, since none of our patients has received a systemic therapy.

Whether OCTs are associated with treatment response to Sorafenib has not been investigated yet. To date, to the best of our knowledge, there exist no data regarding the clinical, prognostic and therapeutical relevance of OCTs in HCC. Because of the impact of OCT1 on tumor characteristics and patient survival we focused on this transporter on protein level. The relevance of OCT3 needs to be further investigated. Future studies will be necessary to evaluate possible diagnostic and therapeutic consequences.

## Conclusions

Downregulation of OCT1 (*SLC22A1*) expression in HCC is associated with advanced tumor stages and a worse patient survival. These findings could be important for treatment options.

## Competing interests

The authors declare that they have no competing interests.

## Authors' contributions

AL, TZ designed research, collected and analyzed data, wrote the paper. AZ, JK, JS, MH, MHL: collected and analyzed data. AL, DF, GO, MH and NW collected tissue samples. MHL performed statistical analysis. AS performed histological evaluation. DG, PRG, MS: made critical review of the manuscript. All authors read and approved the final manuscript.

## Pre-publication history

The pre-publication history for this paper can be accessed here:

http://www.biomedcentral.com/1471-2407/12/109/prepub
